# Astrocytic expression of Parkinson's disease-related A53T α-synuclein causes neurodegeneration in mice

**DOI:** 10.1186/1756-6606-3-12

**Published:** 2010-04-21

**Authors:** Xing-Long Gu, Cai-Xia Long, Lixin Sun, Chengsong Xie, Xian Lin, Huaibin Cai

**Affiliations:** 1 Units of Transgenesis, Laboratory of Neurogenetics, National Institute on Aging, National Institute of Health, Bethesda, Maryland 20892, USA

## Abstract

**Background:**

Parkinson's disease (PD) is the most common movement disorder. While neuronal deposition of α-synuclein serves as a pathological hallmark of PD and Dementia with Lewy Bodies, α-synuclein-positive protein aggregates are also present in astrocytes. The pathological consequence of astrocytic accumulation of α-synuclein, however, is unclear.

**Results:**

Here we show that PD-related A53T mutant α-synuclein, when selectively expressed in astrocytes, induced rapidly progressed paralysis in mice. Increasing accumulation of α-synuclein aggregates was found in presymptomatic and symptomatic mouse brains and correlated with the expansion of reactive astrogliosis. The normal function of astrocytes was compromised as evidenced by cerebral microhemorrhage and down-regulation of astrocytic glutamate transporters, which also led to increased inflammatory responses and microglial activation. Interestingly, the activation of microglia was mainly detected in the midbrain, brainstem and spinal cord, where a significant loss of dopaminergic and motor neurons was observed. Consistent with the activation of microglia, the expression level of cyclooxygenase 1 (COX-1) was significantly up-regulated in the brain of symptomatic mice and in cultured microglia treated with conditioned medium derived from astrocytes over-expressing A53T α-synuclein. Consequently, the suppression of COX-1 activities extended the survival of mutant mice, suggesting that excess inflammatory responses elicited by reactive astrocytes may contribute to the degeneration of neurons.

**Conclusions:**

Our findings demonstrate a critical involvement of astrocytic α-synuclein in initiating the non-cell autonomous killing of neurons, suggesting the viability of reactive astrocytes and microglia as potential therapeutic targets for PD and other neurodegenerative diseases.

## Background

α-synuclein (α-syn) is a major component of Lewy bodies (LB) and Lewy neurites (LN) appearing in the postmortem brain of Parkinson's disease (PD) and other synucleinopathies [1,2]. Genetic mutations in α-syn, including point mutations (A53T, A30P and E46K) and multiplications have been linked to familial PD and Dementia with LB [3-7]. Although the precise function of α-syn remains elusive, overwhelming evidence indicates that malfunction of α-syn, especially the aggregation of misfolded α-syn, plays an important role in the process of neurodegeneration [5,8].

Neuronal expression of either human wild-type or PD-related mutant α-syn induces neurodegeneration associated with pathological accumulations of α-syn and reactive astrogliosis [9-13]. In addition, α-syn-containing inclusion bodies are present in oligodendrocytes of multiple system atrophy (MSA) [14]. Transgenic mice over-expressing wild-type α-syn in oligodendrocytes display severe neurological alterations and neurodegeneration [15,16]. Previous studies also reveal that α-syn-containing inclusion bodies present in astrocytes of sporadic PD [17-19] and over-expression of wild-type or C-terminally truncated α-syn in U373 astrocytoma cells induces apoptotic death of astroglial cells [20]. However, whether the astrocytic expression of PD-related A53T α-syn contributes to neurodegeneration is unknown [21].

Here we generated a new line of α-syn inducible transgenic mice in which the PD-related A53T α-syn was selectively expressed by astrocytes. Interestingly, the mutant mice developed rapidly progressed paralysis, likely resulting from widespread astrocytosis, severe microglial activation, and especially midbrain dopaminergic and spinal cord motor neuron degeneration. These results demonstrate that α-syn-mediated cytotoxicity to astrocytes is critical for inducing the non-cell autonomous degeneration of neurons, suggesting that the maintenance of normal function of astrocytes is important in ameliorating the progression of neurodegeneration in PD and other neurodegenerative diseases.

## Results

### Generation of human *α-synuclein *inducible transgenic mice that selectively express exogenous α-*synuclein *in astrocytes

**Figure 1:**
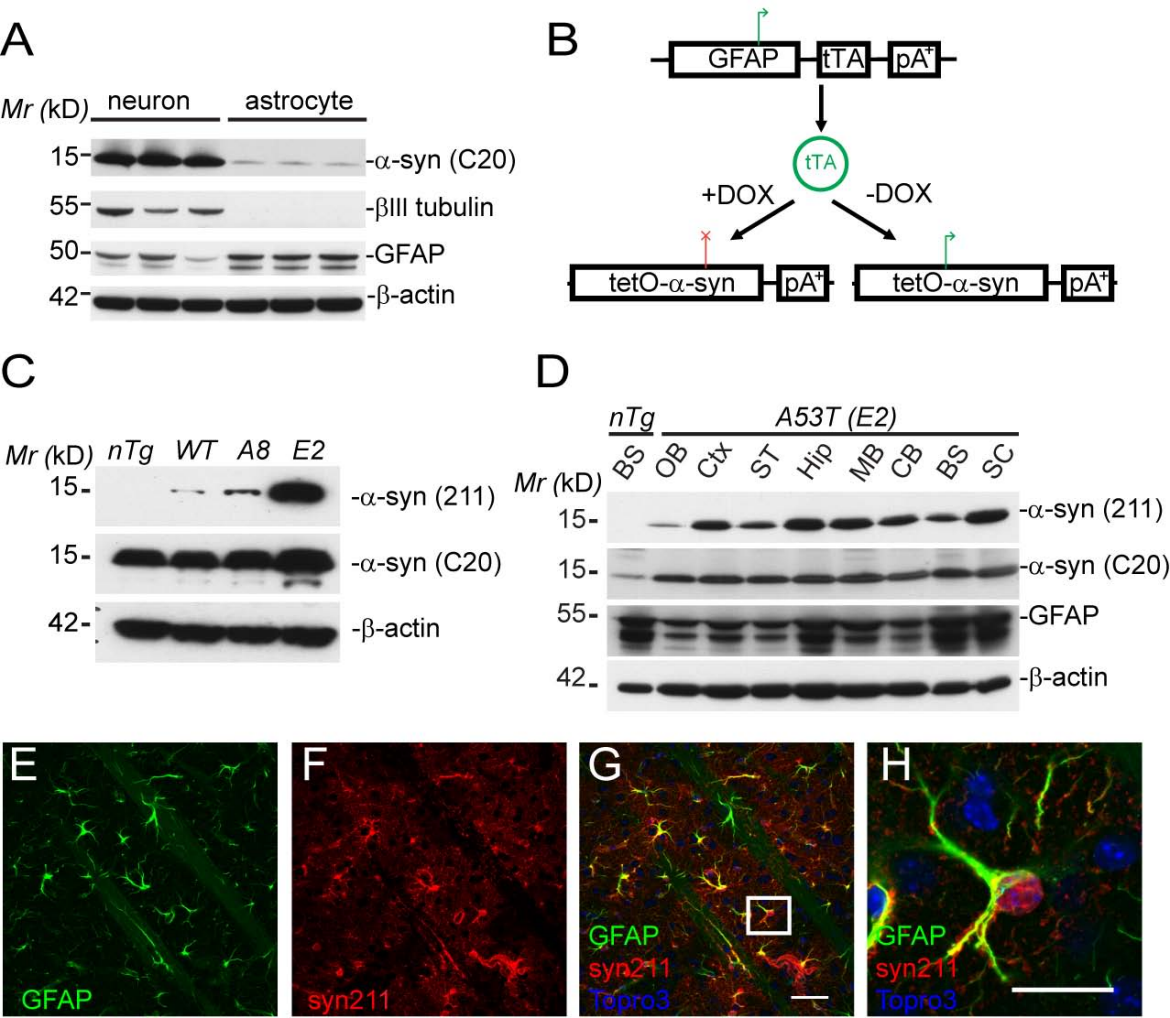
**Astrocytic expression of human *α-synuclein *in mice**. **(A)**. Western blot analysis of endogenous α-syn expression in primary cultured cortical neurons and astrocytes. Protein extracts (8 μg) from cultured cortical neurons and astrocytes were immunoblotted using antibodies against human/mouse α-syn (C20), β III-tubulin (neuronal marker), and GFAP (astrocyte maker). β-actin serves as the loading control. **(B) **A schematic outline of the "tet-off" system for astrocytic expression of α-*syn *in mice. The tetracycline operator-controlled human α-*syn *responder mice (*tetO-α-syn*) were crossed with *GFAP-tTA *activator mice to generate *GFAP-tTA/tetO-α-syn *double transgenic (*A53T*) mice, which selectively expressed exogenous human α-*syn *in astrocytes. **(C) **Western blot analysis of human α-syn expression in transgenic mice. One wild-type (*WT*) and two *A53T *lines (*A8 *and *E2*) of transgenic mice were obtained. The expression of exogenous human α-syn was detected by a human α-syn-specific antibody, α-syn (211). **(D) **The expression pattern of human α-syn in the brain of *A53T *(*E2 *line) transgenic mice revealed by Western blot using both human-specific and human/mouse α-syn antibodies. BS: brainstem, OB: olfactory bulb, Ctx: cortex, ST: striatum, Hip: hippocampus, MB: midbrain, CB: cerebellum; SC: spinal cord. **(E-H) **Representative images show human α-syn (red) and GFAP (green) co-staining in the striatum of *A53T *mice. The α-syn immunoreactivity is restricted to GFAP-expressing astrocytes. Scale bar: 20 μm.

α-*syn *is most abundant in neurons [22]. However, the expression of α-*syn *in astrocytes has not been rigorously examined. Here we prepared the astrocytic and neuronal cell cultures from postnatal day 1 pups and compared the expression of α-*syn *in these cells. Consistent with previous findings [22], α-syn was highly expressed in neuronal cultures (Fig. 1A). Interestingly, a lower level expression of α-syn was consistently detected in astrocytes (Fig. 1A).

To reveal the potential contribution of astrocytic expression of α-*syn *in the pathogenesis of PD, we generated new lines of α-*syn *inducible transgenic mice that selectively expressed the human wild-type (WT) or PD-related A53T α-*syn *in astrocytes. As outlined in Fig. 1B, we crossbred the human glial fibrillary acidic protein (GFAP) promoter-controlled tetracycline transactivator (*GFAP-tTA*) transgenic mice [23] with the tetracycline operator (tetO)-regulated α-*syn *transgenic mice (*tetO-WT *or *tetO-A53T*) to generate the *GFAP-tTA/tetO-WT *(*WT*) and *GFAP-tTA/tetO-A53T *double transgenic (*A53T*) mice. The expression of exogenous α-syn in the double transgenic mice was demonstrated by Western blot using an antibody specifically against human α-syn. We have obtained one *WT *and two *A53T *lines of transgenic mice (Fig. 1C). Since most of studies focused on the *A53T-E2 *line, we refer this line as *A53T *mice in later description. The *A53T *transgenic mice displayed significant elevation of α-syn expression throughout the CNS, including olfactory bulb, cerebral cortex, striatum, midbrain, hippocampus, cerebellum, brainstem and spinal cord (Fig. 1D). Using an α-syn antibody that recognizes both mouse and human α-syn, Western blot analysis revealed about 10-fold increase of α-syn expression in the brain of *A53T *mice as compared to non-transgenic (*nTg*) littermate controls (see Additional file 1A). Moreover, at the cellular level, the expression of A53T α-syn was restricted to GFAP-positive astrocytes as demonstrated by co-staining of human α-syn with GFAP but not other cell type markers in the brain sections of *A53T *mice (Figs. 1E-H; Additional file 1B-D).

### The *A53T *transgenic mice developed an early onset rapidly progressed movement disability

**Figure 2:**
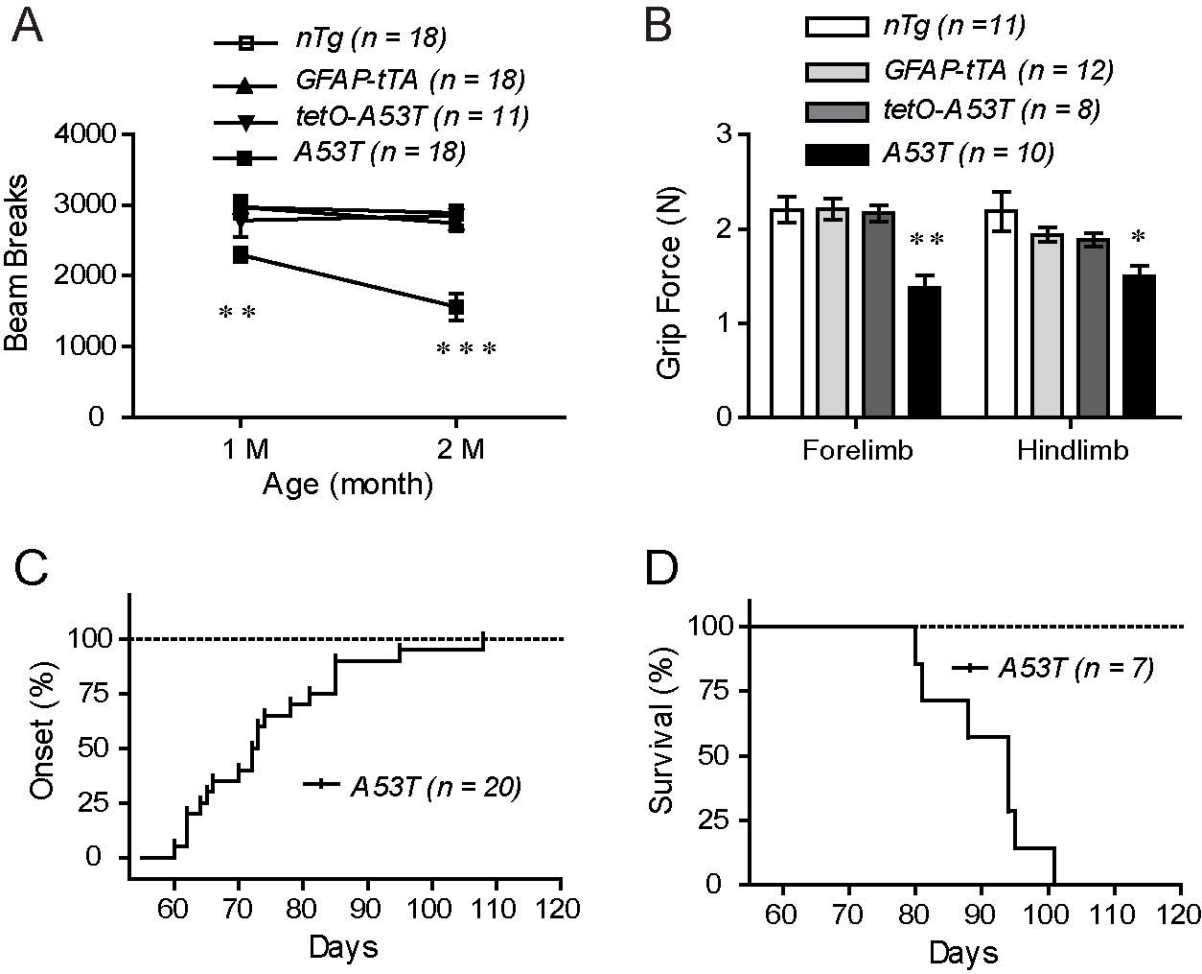
**Early-onset neurological dysfunction and shortened lifespan of *A53T *mice**. **(A) **Open-field tests reveal a significant decline of spontaneous ambulatory activities in *A53T *mice as compared to littermate controls at 1 and 2 months of age. **p < 0.01, ***p < 0.001. **(B) **Both fore and hind limb grip strength of *A53T *mice are impaired compared to littermate controls at 2 months of age. *p < 0.05, **p < 0.01. **(C) **Line graph reveals the onset of paralysis observed in *A53T *mice (n = 20). The average age of onset was 71.6 ± 2.0 days. **(D) **Line graph depicts the survival rate of *A53T *mice (n = 7). The average lifespan of the *A53T *mice was 90.4 ± 2.9 days.

To investigate whether over-expression of A53T *α-syn *in astrocytes caused any behavioral abnormalities, we monitored the body weight and examined the spontaneous locomotor activities and motor coordination of *A53T *mice. The *A53T *mice were viable but appeared smaller and weighted significantly less compared to age-matched *nTg*, as well as *GFAP-tTA *and *tetO-A53T *single transgenic mice at one and two months of age (see Additional file 2A-B). The spontaneous locomotor activities of *A53T *mice measured by the Open-field test were progressively declined from one month to two months of age compared to control mice (Fig. 2A; Additional file 2C-D). The grip strength of *A53T *mice was also significantly reduced in both fore and hind limbs at two months of age (Fig. 2B). The motor coordination of two-month old *A53T *mice seemed, however, not affected in the Rotarod test (see Additional file 2E). After two and a half months of age, the progression of movement disability of *A53T *mice was greatly accelerated. One of the four limbs paralyzed with the average age of onset at 71.6 ± 2.0 days (Fig. 2C, Additional files 3, Additional file 4). The paralysis quickly spread to the remaining limbs, and the affected mice experienced difficulties in eating and drinking after showing paralysis in two or more limbs. As a result, the body weight of symptomatic mice continued to drop during the disease progression (see Additional file 2F), and eventually, the animals died with the average lifespan at 90.4 ± 2.9 days (Fig. 2D, Additional file 5). The penetrance of paralysis was 100% in *A53T *mice.

Previous studies also indicated that GFAP is expressed in neuronal precursor cells during development [24,25]. To assess the impact of developmental expression of A53T *α-syn *on the neurological dysfunction, a cohort of *A53T *mice were administered with doxycycline (DOX), a tetracycline derivative, to block the expression of A53T *α-syn *from embryonic stages to postnatal day 21 (P21). The DOX-exposed *A53T *mice behaved normally at one and two months of age (see Additional file 6). However, two months after removal of DOX, 43% of these mice started to lose body weight and displayed less spontaneous locomotor activities (see Additional file 6). All of these DOX-treated *A53T *mice eventually progressed to complete paralysis with an average age of onset at 102.1 ± 2.8 days and average lifespan at 110.0 ± 3.8 days. These data suggest that the paralysis developed by *A53T *mice is not dependent on the developmental expression of A53T *α-syn*.

### Astrocytic expression of A53T α-*synuclein *induced widespread astrogliosis

**Figure 3:**
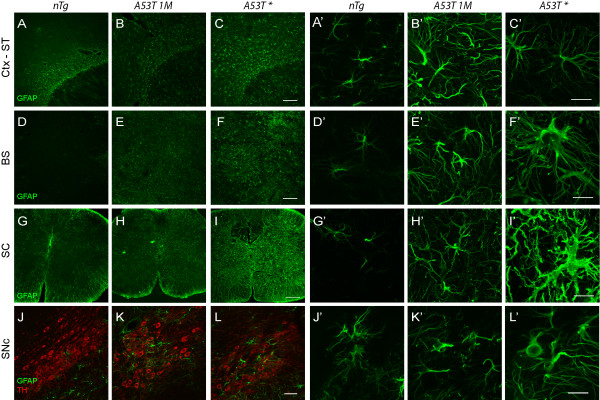
**More GFAP-enriched and hypertrophic astrocytes in *A53T *mice**. (**A-L'**) Representative images show GFAP staining (green) in the cerebral cortex-striatum **(A-C')**, brainstem **(D-F')**, spinal cord **(G-I')**, and substantia nigra pars compacta (SNpc) **(J-L') **of *nTg *mice (2.5-month old) and *A53T *mice at asymptomatic (1-month old, *A53T 1M*) and symptomatic (A53T*) stages. Hypertrophy of astrocytes is shown in the brainstem **(F')**, spinal cord **(I')**, and SNpc **(L') **of symptomatic *A53T *mice. The SNpc dopaminergic neurons were revealed by tyrosine hydroxylase (TH) staining (**J-L**, red). Scale bar: 200 μm **(A-C, D-F, G-I)**, 50 μm **(J-L)**, and 20 μm **(A'-C', D'-F', G'-I', J'-L')**.

To reveal the cause of neurological dysfunction, we checked whether A53T *α-syn *affected astrocytes in the brain. Brain sections of both asymptomatic (*A53T 1M*) and symptomatic mice (A53T*) were examined for GFAP staining (Fig. 3). The number of GFAP-positive astrocytes was dramatically increased in the gray matter of cerebral cortex (Figs. 3B-C), striatum (Figs. 3B-C), brainstem (Figs. 3E-F), spinal cord (Figs. 3H-I), midbrain (Figs. 3K-L), and other brain areas of *A53T *mice as compared to age-matched *nTg *mice (Figs. 3A, D, G and 3J). The increase of GFAP expression was also confirmed by quantitative RT-PCR and Western blot analyses in which the level of GFAP expression was further elevated in the symptomatic mice (see Additional file 7). Moreover, the soma size of GFAP-positive astrocytes was also significantly increased in the brainstem, spinal cord, and midbrain of symptomatic *A53T *mice (*nTg*, 27.8 ± 5.7 μm^2^; A53T*, 132.0 ± 34.9 μm^2^; p = 0.0051) (Figs. 3A'-C', D'-F', G'-I' and 3J'-L'). Taken together, these results demonstrate that exogenous expression of human A53T *α-syn *in astrocytes induced severe astrogliosis in mice.

### Astrocytic expression of A53T α-*synuclein *disrupted the normal function of astrocytes

**Figure 4:**
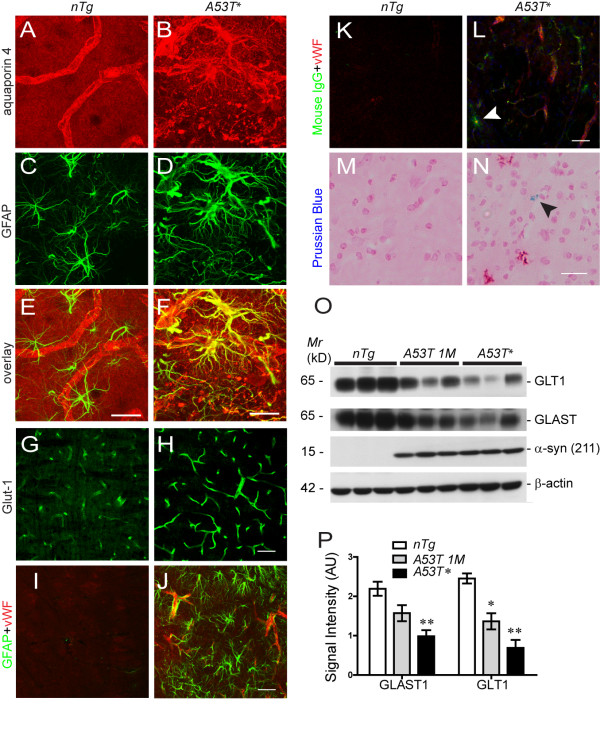
**Dysfunction of astrocytes in *A53T *mice**. **(A-F) **Representative images show the co-staining of GFAP **(C, D, green) **and aquaporin 4 **(A, B, red) **in the brainstem of symptomatic *A53T *mice **(B, D, F) **and *nTg *littermates **(A, C, E)**. Aquaporin 4 was redistributed into the cell body and proximal processes of astrocytes in the symptomatic *A53T *mice **(B, D, F)**. Scale bar: 20 μm. **(G-H) **Representative images show glucose transporter 1 (Glut-1) staining in the epithelia cell of the symptomatic *A53T *mice **(H) **as well as age-matched *nTg *controls **(G)**. The expression of Glut-1 was up-regulated in the *A53T *mice. Scale bar: 50 μm. **(I-J) **Double labeling of GFAP (green) and von Willebrand factor (vWF, red) in the brainstem of symptomatic *A53T *mice **(J) **and *nTg *littermates **(I)**. The expression of vWF was elevated in *A53T *mice. Scale bar: 50 μm. **(K-L) **Representative images show double staining for IgG (green, arrowhead) and vWF (red) in the brainstem of symptomatic *A53T *mice **(L) **but not in the *nTg *littermate controls **(K)**. Scale bar: 50 μm. **(M-N) **Representative images show the Prussian blue staining (nuclei were counterstained with fast red) in the brain stem of symptomatic *A53T (N) *and control mice **(M)**. Scale bar: 20 μm. **(O) **Western blot analysis of the expression levels of excitatory amino acid transporter 1 and 2 (GLAST and GLT1) in the brainstem of *A53T *and control *nTg *mice. **(P) **Bar graph compares the levels of GLT1 and GLAST proteins in *nTg*, asymptomatic (*A53T 1M*) and symptomatic *A53T *mice (n = 3 per genotype). *p < 0.05, **p < 0.01.

Astrocyte plays an important role in maintenance the homeostasis of extracellular environment of neurons [26]. The processes of astrocyte end-feet encircle the endothelial cell of blood vessels for maintenance of blood-brain barrier (BBB) and withdrawal of nutrients from the blood [27,28]. The astrocyte-endothelial cell interaction plays a major role in regulating brain water and electrolyte metabolism under both normal and pathological conditions [27]. In search for the signs of BBB damage in the brain of symptomatic *A53T *mice, we examined the expression and subcellular distribution of aquaporin 4, glucose transporter 1 (Glut1) and von Willebrand factor (vWF) in astrocyte and vascular endothelial cells. As shown in the brainstem of *nTg *mice, aquaporin 4 was normally confined to the end-foot of astrocytes that outlined the blood vessels in the brain (Figs. 4A, C and 4E). In the brainstem and spinal cord of symptomatic *A53T *mice; however, aquaporin 4 was redistributed to the soma and proximal processes of astrocytes (Figs. 4B, D and 4F). The redistribution of aquaporin 4 to the soma of astrocyte has been shown before to impair the shielding of end-foot around blood vessels [29]. In addition, abnormal accumulation of Glut1 and vWF in the vascular endothelial cell was observed at the brainstem and spinal cord of symptomatic *A53T *mice (Figs. 4G-J), indicating the dysfunction of vascular endothelial cells. Together these data indicate that astrocytic expression A53T *α-syn *may cause damage to BBB. In support of this notion, focal deposition of immunoglobulin (Ig) G was observed in the brainstem of *A53T *symptomatic mice (Fig. 4L). Moreover, Prussian blue staining from symptomatic mice identified deposits of hemosiderin, a hemoglobin derivative, in the brainstem (Fig. 4N), suggesting the occurrence of microhemorrhage in these mice.

Astrocytes also express excitatory amino acid transporters, which are responsible for the majority of glutamate uptake in the brain and its dysfunction has been associated with multiple psychiatric and neurological disorders [30]. Here we found that the expression of excitatory amino acid transporter 1 and 2 (GLAST, GLT1) was significantly decreased in asymptomatic and symptomatic *A53T *mice (Figs. 4O-P), indicating that excitotoxicity may occur in *A53T *mice.

### Astrocytic expression of A53T α-*synuclein *induced activation of microglia in the CNS

**Figure 5:**
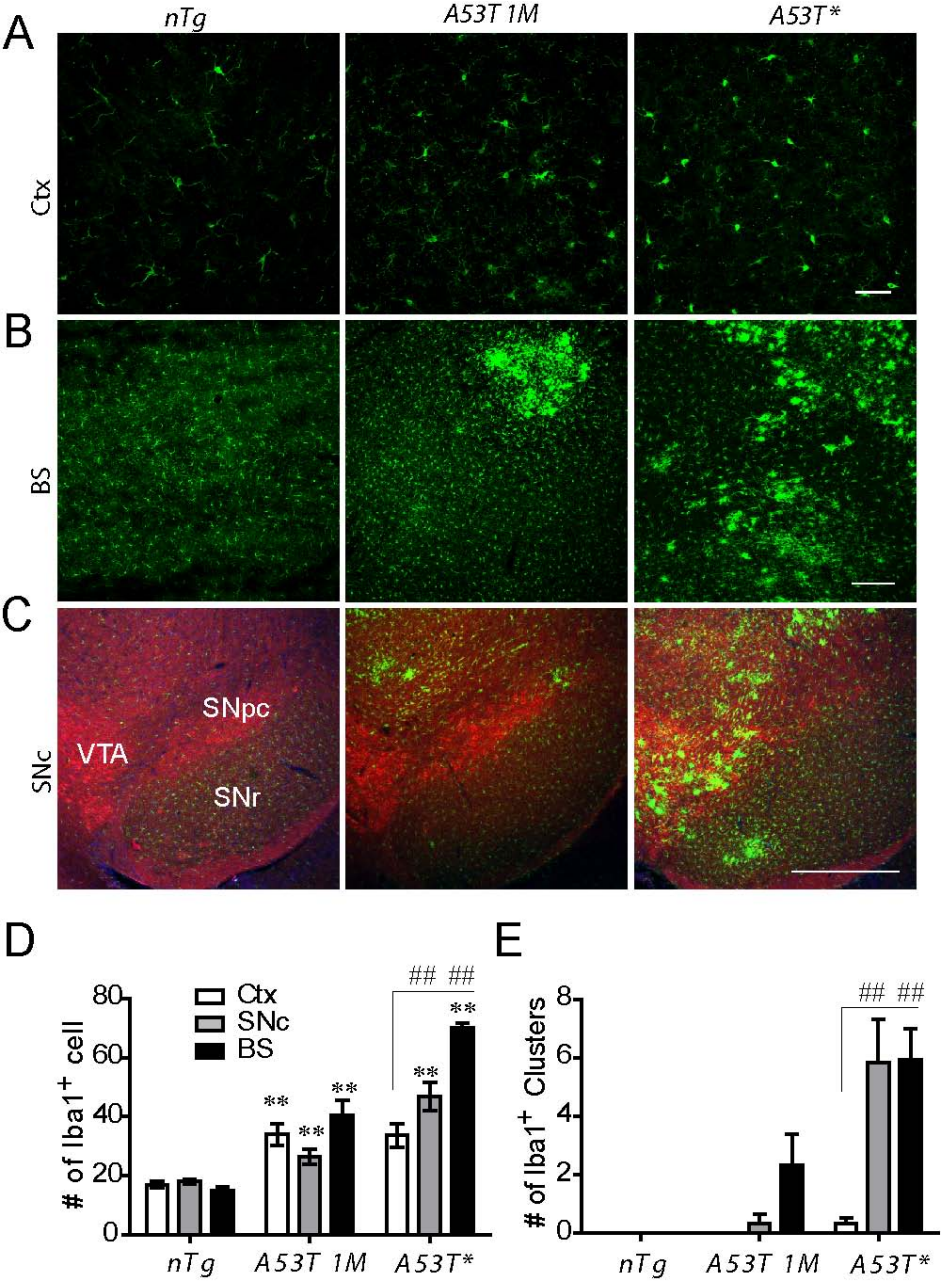
**Activation of microglia in *A53T *mice**. **(A-C) **Representative images show Iba1 staining in cortex (Ctx), brainstem (BS) and substance nigra pars compacta (SNc) sections derived from *A53T *and *nTg *littermate control mice. Scale bars: 50 μm **(A)**, 200 μm **(B-C)**. **(D-E), **Quantitative measurement of the microglia (Iba1 positive cells) and microglia clusters in the cortex, substantia nigra pars compacta and brainstem (n = 3 per genotype). **p < 0.01, ***p < 0.001, compared with *nTg*; ^##^p < 0.01, compared with cortex.

Astrocytes are active players in cerebral innate immunity [31]. To investigate whether reactive astrocyte in symptomatic *A53T *mice triggered inflammatory response, we examined whether microglia, the immune defense cell in the CNS, were activated. Progressive activation of microglia was observed in the brain of *A53T *mice (Figs. 5A-C) as evidenced by enlarged soma and by the appearance of microglia clusters (Figs. 5A-C). Clusters of activated microglia with large, amoeboid shape and fewer branches were spotted firstly in the peduncle of cerebellum at asymptomatic stage, and later in the brainstem (Figs. 5B and 5D-E) and midbrain (Figs. 5C and 5D-E) at symptomatic stage. Although astrocytosis was observed in the forebrain regions (Fig. 3), interestingly, no obvious activation of microglia was observed in the cortical areas (Figs. 5A and 5D-E). Consistent with the morphology change of the microglia, the expression of Iba1 was dramatically up-regulated in the *A53T *mice as evidenced by quantitative RT-PCR and western blot analysis (see Additional file 7). More interestingly, clusters of activated microglia were found adjacent to spinal motor neurons and dopaminergic neurons in substantial nigra pars compacta (SNpc) (see Additional file 8). These data indicate that activated microglia may attack neurons in the spinal cord and SNpc and lead to neurodegeneration.

### Astrocytic expression of A53T α-*synuclein *caused neurodegeneration

**Figure 6:**
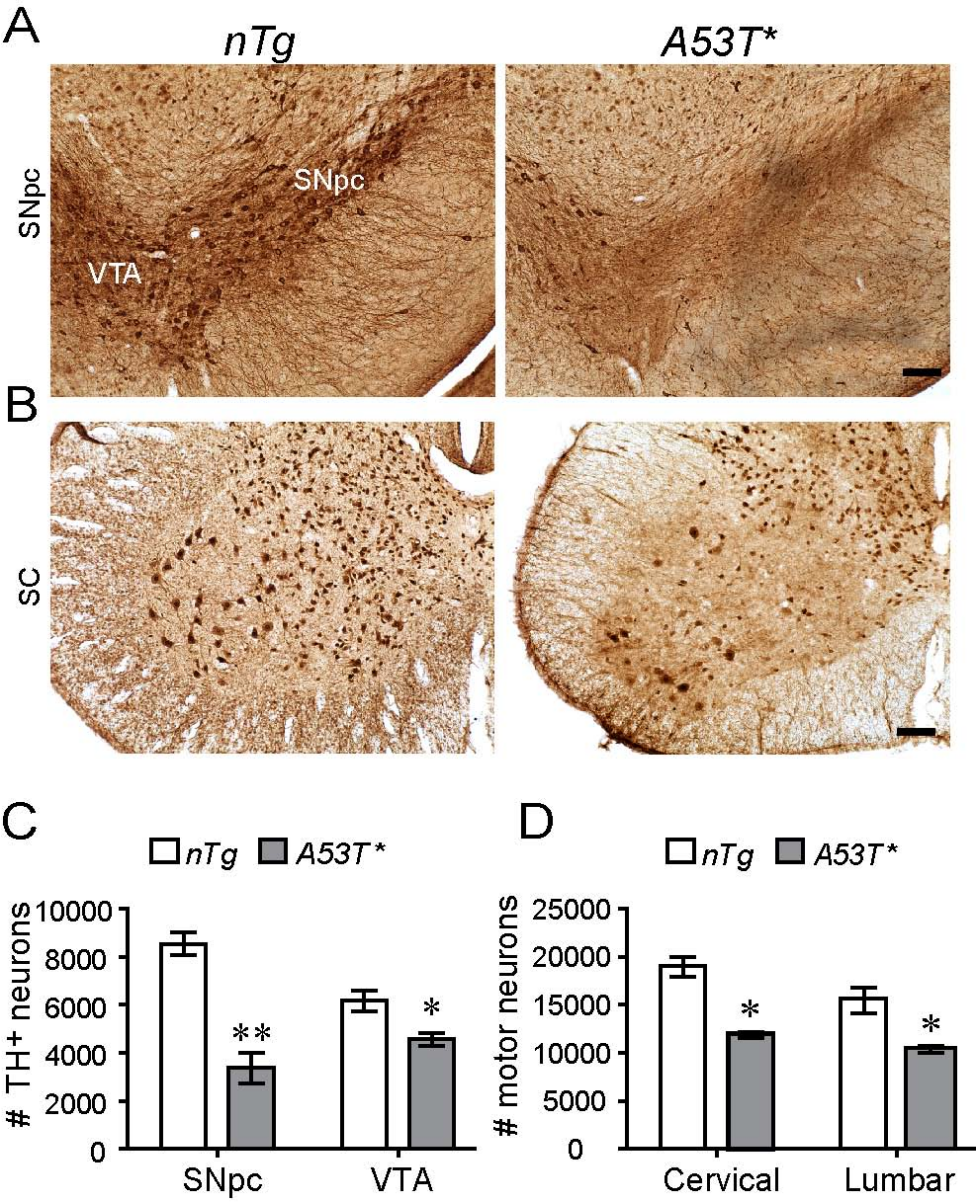
**Neuron loss occurred in the midbrain and the spinal cord of symptomatic *A53T *mice**. **(A) **Representative images show TH and NeuN double staining in the SNpc area of symptomatic *A53T *mice and age-matched *nTg *littermates. Scale bars: 100 μm. **(B) **Representative images show NeuN staining in the ventral horn of lumbar spinal cord of symptomatic *A53T *mice and age-matched *nTg *littermates. Scale bar: 100 μm. **(C) **Bar graph depicts the numbers of TH-positive dopaminergic neurons in the SNpc and ventral tegmental area (VTA) of symptomatic *A53T *mice and *nTg *littermates (n = 3) estimated by unbiased stereological methods. *p < 0.05, **p < 0.01. **(D) **Bar graph reveals the numbers of motor neurons remained in the cervical and lumbar spinal cord of the symptomatic *A53T *mice and age-matched *nTg *littermates (n = 3). *p < 0.05.

To examine whether neuronal loss occurred in the brain of symptomatic *A53T *mice, unbiased stereological analysis was employed to estimate the remained neurons in the cortex, striatum, SNpc, and spinal cord. While the numbers of tyrosine hydroxylase (TH)-positive dopaminergic neurons in SNpc and ventral tegmental area (VTA) were not altered in the *A53T *mice at 1 month of age (see Additional file 9), they were decreased by 60.5% in SNpc and by 26.1% in VTA of symptomatic *A53T *mice as compared with age-matched *nTg *littermates (Figs. 6A, C). Similarly, the numbers of motor neurons were significantly reduced in both cervical and lumber spinal cord of symptomatic *A53T *mice compared to control mice (Figs. 6B, D). In contrast, the number of neurons in the cerebral cortex and striatum area symptomatic *A53T *mice remained unchanged (see Additional file 9). Taken together, significant neuronal loss was detected in the midbrain and spinal cord of *A53T *symptomatic mice, which may underlie the movement disability developed in these animals. In addition, the close correlation of regional distribution between the occurrence of neurodegeneration and microglial activation indicate that activated microglia may play an important role in triggering the loss of neuron.

### Microglia-mediated inflammatory responses were involved in the neurodegeneration of *A53T *mice

**Figure 7:**
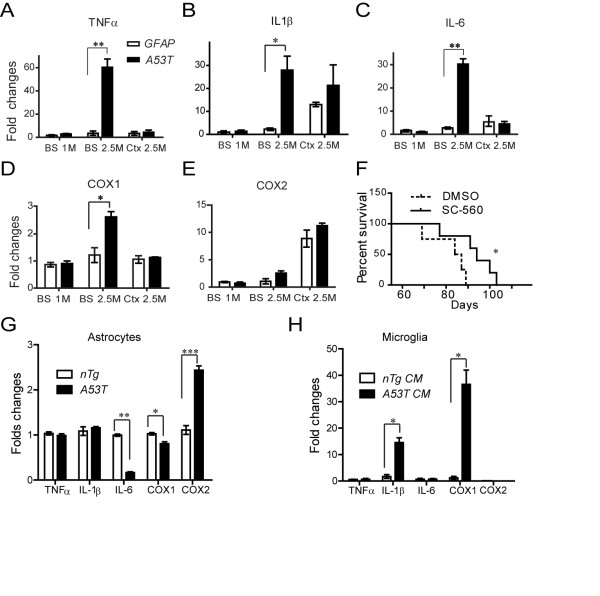
**Increase of inflammatory response in *A53T *mice**. **(A-E), **Quantitative PCR array analysis of the expression of cytokines in the tissues of the brainstem (BS) and cerebral cortex (Ctx) of *A53T *mice at asymptomatic (1 M) and symptomatic (2.5) stages (n = 3 per genotype and per stage). *p < 0.05, **p < 0.01. **(F), **Survival curve shows the lifespan of *A53T *mice treated with COX-1 inhibitor, SC-560 (n = 5) or 40% vehicle, DMSO (n = 4). Log-rank test, *p < 0.05. (**G**), Quantitative PCR array analysis of the expression of cytokines in cultured astrocytes from the brainstem of *A53T *or *nTg *mice (n = 3 per genotype). *p < 0.05, **p < 0.01, ***p < 0.001. (**H**), Quantitative PCR array analysis of the expression of cytokines in the cultured primary microglia treated with conditioned medium from cultured astrocytes of the brainstem of *A53T *or *nTg *mice (n = 3 per genotype). *p < 0.05.

Increasing studies demonstrate the involvement of inflammation in the degeneration of neurons [32]. Since inflammation is often in accompany with increased expression of cytokines, such as tumor necrosis factor-α (TNF-α), interleukin-1β (IL-1β), and interleukin-6 (IL-6), we quantified the expression of TNF-α, IL-1β, and IL-6 in the cortex and brainstem of *A53T *mice at both presymptomatic and symptomatic stages. At the presymptomatic stage when little activated microglia were observed, there was no significant alteration of TNF-α, IL-1β, and IL-6 expression in the cerebral cortex and brainstem of *A53T *mice (Figs. 7A-C). By contrast, at the symptomatic stage, a dramatic increase of TNF-α, IL-1β, and IL-6 expression was observed in the brainstem but not in the cerebral cortex of *A53T *mice (Figs. 7A-C), which is closely correlated with the presence of activated microglia (Figure 5). These results indicate that the increase production of cytokines is mainly from activated microglia. Consistently, the expression level of cyclooxygenase-1 (COX-1), which expression is restricted to microglia, was also up-regulated in the brainstem of symptomatic *A53T *mice; while the expression level of neuron-enriched COX-2 was not affected (Figs. 7D-E).

To further demonstrate the origin of cytokines from activated microglia, we compared the expression of TNF-α, IL-1β, and IL-6 in primary cultured astrocytes from the brainstem of symptomatic *A53T *mice and age-matched littermate controls. No significant increase of TNF-α, IL-1β, and IL-6 expression was observed in cultured astrocytes from *A53T *mouse brainstem compared to control cultures (Fig. 7G), indicating a limited contribution of astrocytes in the direct production of cytokines. In contrast, the expression level of IL-1β and COX-1 was significantly up-regulated in cultured microglia treated with conditioned medium derived from cultured astrocytes of *A53T *mouse brainstem (Fig. 7H). These results indicate that substances released from A53T α-*syn*-expressing astrocytes may induce the production of proinflammatory cytokines from microglia.

COX-1 is the key and rate-limiting enzyme in conversion of arachidonic acid to prostaglandins (PGs) and plays an important role in the neuroinflammatory process [33]. To evaluate the contribution of COX-1 activity in the progression of paralysis in *A53T *mice, we treated presymptomatic 2-month old *A53T *mice with SC-560 (30 mg/kg, i.p.), a selective inhibitor of COX-1 [34] once a day for 7 days. Compared to that of vehicle-treated mice, the lifespan of SC-560-treated *A53T *mice was significantly extended (Fig. 7F, p = 0.035), suggesting that microglia-mediated inflammatory responses directly contribute to the neurodegeneration in *A53T *mice.

### The accumulation of aggregated and truncated forms of α-synuclein in *A53T *mice

**Figure 8:**
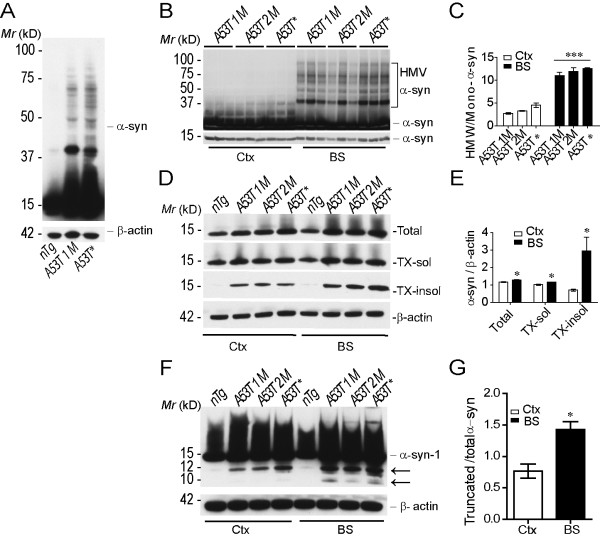
**Increased accumulation of aggregated and truncated α-synuclein in *A53T *mice**. **(A) **Western blot analysis shows high molecular weight (HMW) α-syn-positive bands in brainstem homogenates of *A53T *mice as compared to *nTg *controls. β-actin serves as loading control. **(B-C) **Western blot analysis shows the presence of HMW α-syn was more abundant in the brainstem than the cerebral cortex of *A53T *mice at different time points (B). Bar graph shows the ratio of HMW-α-syn/monomeric (Mono)-α-syn in the cortex (Ctx) and brainstem (BS) of *A53T *mice during the progression of paralysis (C). ***p < 0.0001. (**D-E**) Western blot analysis shows the level of α-syn in sequentially detergent-extracted cerebral cortex and brainstem homogenates of *nTg *and *A53T *mice (D). Bar graph reveals the ratio of total, TX-soluble, and TX-insoluble α-syn in the Ctx and BS of symptomatic *A53T *mice (E). Signal intensities of α-syn were normalized by β-actin. *p < 0.05. **(F-G) **Western bolt analysis shows the level of C-terminal truncated α-syn (pointed by arrows) using an antibody that specifically recognizes N-terminal 1-100 amino acids of α-syn (F). Bar graph depicts the ratio of truncated α-syn against total α-syn in the Ctx and BS of symptomatic *A53T *mice (G). *p < 0.05.

The aggregated or truncated forms of α-syn has been indicated in initiating the downstream pathogenic events of neurodegeneration [35]. We decided to examine the correlation between each form of α-syn and the progression of paralysis in *A53T *mice. We first checked the presence of α-syn aggregates in the brain, which appeared as α-syn-positive high molecular weight bands in Western blot analysis and resistant to Triton-X100 (TX) extraction in tissue fractionation studies. The aggregation of α-syn was significantly increased in the brain of symptomatic *A53T *mice compared with *nTg *controls and asymptomatic mice (Fig. 8A). Moreover, in agreement with the above neuropathological studies, the ratios of TX-insoluble α-syn were significantly higher in the brainstem as compared to the cerebral cortex of *A53T *mice (Figs. 8B-E). A similar phenomenon was also observed for truncated form of α-syn, which was selectively up-regulated in the brainstem of symptomatic *A53T *mice (Figs. 8F-G). Taken together, these data suggest that the aggregated and truncated forms of α-syn may impair the normal function of astrocytes and initiate the downstream pathogenic cascades leading to the loss of neurons in *A53T *mice.

## Discussion

Although α-syn is less abundant in glial cells [36], α-syn-containing inclusion bodies are present in astrocytes of postmortem PD brains [17-19]. By using a "tet-off" inducible transgenic approach, we selectively expressed PD-related A53T α-*syn *in astrocytes to investigate the pathological consequence of astrocytic expression and aggregation of α-syn *in vivo*. We show here that the presence of excess A53T α-syn in astrocyte caused severe astrogliosis, which disrupted the normal function of astrocytes critical for maintaining the integrity of blood-brain barrier and homeostasis of extracellular glutamate. The A53T *α-syn *induced astrogliosis also led to inflammation and microglial activation, particularly in the midbrain, brainstem and spinal cord. We further revealed a significant loss of dopaminergic neurons in the midbrain and motor neurons in the spinal cord of symptomatic mice, which may underlie the paralysis phenotypes developed by these mutant mice. In addition, COX-1-mediated inflammatory pathways may contribute to the neurodegeneration as evidenced by the beneficial effect of COX-1 inhibitor in extending the lifespan of *A53T *mice. Finally, the more severe pathological abnormalities shown in the brainstem of *A53T *mice were correlated with the more abundant accumulation of aggregated as well as truncated forms of α-syn in this region. Together, our findings demonstrate that astrocytic expression of PD-related A53T α-synuclein causes non-cell autonomous killing of neurons implicated in PD and motor neuron diseases, suggesting that reactive astrocytes may serve as a potential therapeutic target for treatment of these movement disorders.

It is rather surprising to find that over-expression of A53T α-*syn *in astrocytes induced more robust loss of midbrain dopaminergic neuron than other published A53T α-*syn *transgenic mice in which the expression of mutant α-*syn *is under the control of neuronal promoters [10,12,37,38]. Astrocytes may have to keep a lower level expression of α-*syn *for their normal functions. Therefore, over-expression of α-*syn *in astrocytes may cause more severe phenotypes compared to that in neurons. It is also possible that astrocyte is less able to detoxify the excess α-*syn *because of its lower level of cathepsin D which is recently proved to effectively degrade α-*syn *[39-41]. To address the importance of expression level of exogenous A53T α-*syn *in astrocytes in determining the onset of behavioral and neuropathological phenotypes in *A53T *mice, we also examined the behavioral and neuropathological phenotypes of *A53T-A8 *mice (the low expression line). Compared to the early onset paralysis exhibited in *A53T-E2 *mice (the high expression line), no obvious behavior abnormalities were observed in *A53T-A8 *mice up to 12 months of age (data now shown). However, astrogliosis and modest microglial activation were detected in the brainstem and SNpc area of *A53T-A8 *mice at 12 months of age (see Additional file 10). The milder phenotypes of *A53T-A8 *mice clearly indicate that the expression level of exogenous α-*syn *determines the onset and severity of observed behavioral and pathological abnormalities in these mutant mice. This observation is also consistent with the earlier genetic studies that multiplications of *α-syn *gene in patients cause early-onset familial PD [3,4].

Astrocytic expression of A53T α-*syn *appears to affect multiple functions of astrocytes, resulting in decreased glutamate transporter expression, and disruption of brain-blood barrier. Astrogliosis happened throughout the brain and spinal cord at both asymptomatic and symptomatic stages. However, the activation of microglia was relatively confined to the midbrain, brainstem and spinal cord of symptomatic mice. It remains to determine why microglia responds differently to the dysfunction of astrocytes in different regions of the brain. Alternatively, under the stress of excess A53T α-*syn*, astrocytes may react differently in the brainstem compared to the cerebral cortex. In line with this notion, more aggregated and truncated forms of α-syn were detected in the brainstem than in the cortex. Astrocytes in the brainstem are perhaps less capable to remove the toxic α-syn species from the cells and are thereby more vulnerable to α-syn-mediated cytotoxicity, which may generate greater damage to surrounding cells, including enhanced activation of microglia and loss of neurons.

The degeneration of midbrain dopaminergic neurons and spinal motor neurons may account for the paralysis of symptomatic *A53T *mice. Although the precise molecular mechanism of this rather selective loss of dopaminergic and motor neurons remains to be elucidated, it is possible that the survival of these neurons is more dependent on the normal function of astrocytes than other types of neurons. For instance, both midbrain dopaminergic neurons and motor neurons are preferentially vulnerable to the dysfunction of glutamate transporters [42], which were also down-regulated in our *A53T *mice. Interestingly, microglial activation was mainly detected in regions where neurodegeneration happened in *A53T *mice. The activated microglia is known to promote neurodegeneration by producing proinflammatory or potentially neurotoxic effectors, including IL-1β, NO, or reactive oxygen species (ROS) [43-45]. Microglia presents the most highest density in SNpc [46]. Thus the loss of SNpc dopaminergic neurons in *A53T *mice may attribute to the activation of nearby microglia. This hypothesis is in line with recent reports that lipopolysaccharide, an activator of microglia, causes SNpc dopaminergic neuron degeneration *in vivo *[47-49]. Nonetheless, it will be interesting to determine which factor is more important in the degeneration of dopaminergic neurons in *A53T *mice.

Our findings firmly support the non-cell autonomous toxicity which is well documented in neurodegenerative disease including PD, amyotrophic lateral sclerosis (ALS), Huntington's disease (HD) and Alzheimer's disease (AD) [50]. α-syn expression in oligodendrocytes causes neuronal degeneration [16], while astrocytes expressing mutant Cu/Zn superoxide dismutase (SOD1) drive disease progression [51]. Recent study shows that mutant huntingtin in glial cells induces HD neurological symptoms even when it is not overexpressed [52], strongly indicating the critical role of mutant huntingtin in glial cells. Given the non-cell autonomous toxicity caused by glial cells in neurodegenerative disease, glial cells in particular reactive astrocytes may serve as a potential therapeutic target for treatment of these movement disorders

## Conclusions

Our studies from this new line of A53T α-*syn *transgenic mice not only provide strong evidences for the critical involvement of astrocytes in the pathogenesis of PD and motor neuron diseases, but also provide a very useful *in vivo *system to test novel therapeutics for preventing the loss of dopaminergic and motor neurons.

## Methods

### Mice

To generate human A53T *α-synuclein *(*α-syn*) inducible transgenic mice, a DNA fragment containing human wild-type (WT) or A53T *α-syn *coding sequence was inserted into a tetracycline operator-regulated gene expression vector (tet-O) to generate the *WT *or *A53T *inducible expression construct. The insert was then purified and microinjected into fertilized oocytes derived from C57BL6/J mice. One *WT *and two *A53T *founders were obtained, which were used to cross with *GFAP-tTA *mice [23] to generate *GFAP-tTA/tetO-α-syn *double transgenic mice. The mice were housed in a pathogen-free climate-controlled facility with *ad libitum *access to regular diet and water. Genotypes were determined by PCR analysis of tail DNA extracted by DirectPCR Reagents (VIAGEN Biotech, CA). PCR primers for genotyping *GFAP-tTA *mice are: tTA-F (5'CCCTTGGAATTGACGAGTAC GGTG3') and MP1R (5'TGGTGTATGAGCGGCGGCGACGGCAG3'); and for genotyping tetO-α-syn mice are: PrpEx2-F (5'TACTGCTCCATTTTGCGTGA3') and SNCA-R (5'TCCAGAATTCCTTCCTGTGG3'). All mouse work follows the guidelines approved by the Institutional Animal Care and Use Committees of the National Institute of Child Health and Human Development.

### Behavioral test

*Rotarod test: *as described previously [53], mice were placed onto a rotating rod with auto acceleration from 0 rpm to 40 rpm in 2 min (San Diego Instruments, San Diego, CA). The length of time the mouse stayed on the rotating rod was recorded.

*Open-field test*: as described previously [53], the ambulatory, fine and rearing activities of mice were measured by the Flex-Field activity system (San Diego Instruments, CA). Flex-Field software was used to trace and quantify mouse movement in the unit as the number of beam breaks per 30 min.

*Grip strength measurement: *as described previously [54], mice were allowed to use their forepaws or hind paws to pull or compress a triangular bar attached to a digital force gauge (Ametek, Largo, FL) set up to record the maximal pulling or compressing force. Five measurements were taken for each animal during each test.

### Histology and Immunohistochemical Analyses

As described previously [54], mice were perfused via cardiac infusion with 4% paraformaldehyde in cold PBS. To obtain frozen sections, brain and spinal cord tissues were removed and submerged in 30% sucrose for 24 h and sectioned at 40 μm thickness with cryostat (Leica CM1950). For paraffin sections, sections at 8 μm thickness were obtained according to standard procedure. Antibodies specific to glial fibrillary acidic protein (GFAP) (1:1000, Sigma-Aldrich USA, St. Louis, MO), ionized calcium binding adaptor molecule-1 (Iba1, 1:1000, Wako Chemicals USA, Richmond, VA), tyrosine hydroxylase (TH, 1:1000, Pel-Freez Biologicals, Rogers, AR), α-synuclein (C20 &211, 1:1000, Santa Cruz Biotech, Santa Cruz, CA), Aquaporin 4 (1:500, Chemicon International, Inc USA, CA), glucose transporter 1 (1:500, Chemicon), von Willebrand Factor (1:500, Dako USA, Carpinteria, CA), iNOS (1:500, Sgima), SMI32 (Sternberger Monoclonal, Lutherville, MD), NeuN (1:1000, Chemicon) were used as suggested by manufacturers. Alexa 488 or Alex 568-conjugated secondary antibody (1:1000, Invitrogen) was used to visualize the staining, and Topro3 (1:1000, Invitrogen) was used for counterstaining the nuclei. Fluorescence images were captured using a laser scanning confocal microscope (LSM 510; Zeiss, Thornwood, NJ). The Images of 100 × objective (bar = 20 μM) were presented as a single optic layer after acquired in *z*-series stack scans at 0.8 μM intervals from individual field. The numbers of microglia and microglia cluster in the images taken from 25 × objective (368 μm × 368 μm) were counted.

### Stereology

According to stereotaxic coordinates of mouse brains (3rd edition, Keith B.J. Franklin and George Paxinos), a series of coronal sections across the striatum (9 sections by every 10^th ^section, Bregma -2.06-1.54 mm), SNpc (7 sections by every 4^th ^section, Bregma -2.70- -3.82 mm), as well as cervical (approximate T1-T6) and lumbar spinal cord (approximate L1-L5, 10 sections by every 12^th ^section) were stained with NeuN plus TH and NeuN, respectively, and visualized using the Vectastain ABC kit (Vector Laboratories, Burlingame, CA). The number of NeuN or TH-positive cells was assessed using Stereo Investigator 8, an unbiased stereological procedure with an optical fractionator (MicroBrightField Inc, Williston, VT). The sampling scheme was designed to have coefficient of error (CE) less than 10% in order to get reliable results. All stereological analyses were performed under the 100 × objective of a Zeiss Axio microscope (Imager A1).

### Primary cell cultures

Primary cortical neuron cultures were conducted as described previously [55] by using postnatal day 1 pups. For cortical astrocyte and microglia cultures [56], the dispersed cells were collected by centrifugation and plated on 75 cm^2 ^flasks in DMEM supplemented with 10% fetal bovine serum (FBS). The cells were pre-incubated at 37°C in a humidified atmosphere of air/5% CO_2 _and the medium was changed first 24 h later and on alternate 3-days thereafter. After a pre-culture period of 8-11 days the cellular debris, microglia were lifted from astrocytes layer by shaking the culture flasks at 190 rpm for 3 h at 30°C. The cells attached to the flask were passed and grown in six-well plates for 3 days until harvest for astrocytes culture. The cells floating in the medium were collected by centrifuge and plated on 6-well plates in DMEM supplemented with 10% FBS for microglial culture. After 24 h incubation, the medium of microglial cultures was switched to conditioned medium from cultured astrocytes.

### Tissue fractionation

As previously described (Xian et al., in press), brain tissues (cerebral cortex, brainstem) were weighed and homogenized with 10 volumes of sucrose buffer (0.32 M sucrose, 1 mM NaHCO_3_, 1 mM MgCl_2_, and 0.5 mM CaCl_2_, plus protease and phosphatase inhibitor cocktails). Lysates were centrifuged at 1, 000 g for 10 min to separate supernatant (S1) and pellet (P1). Protein concentrations in S1 were measured by BCA (Pierce Biotechnology, Rockford, IL). S1 contains total α-synuclein protein, representing the sucrose fraction. An aliquot of S1 was diluted in the same volume of Triton extraction buffer (2% Triton X-100, 20 mM HEPES, plus protease and phosphatase inhibitor cocktails), homogenized by sonication, and centrifuged at 20, 000 × g for 30 min to obtain the Triton X-100-soluble (TX-sol) supernatant (S2) and Triton X-100-insoluble (TX-insol) pellet (P2). P2 was washed 4 times by 1% Triton X-100 buffer and centrifuged at 20, 000 × g for 10 min. The pellet fraction was further extracted in 2% SDS buffer (2% SDS, 20 mM HEPES, plus protease and phosphatase inhibitor cocktails) by sonication and centrifuged at 20, 800 × g for 5 min. The supernatant (S3) were present as Triton X-100-insoluble (TX-insol) or SDS-soluble fraction.

### Western Blot

Proteins were size-fractioned by 4-12% NuPage BisTris-polyacrylamide gel electrophoresis (Invitrogen) using MOPS running buffer (Invitrogen), and transferred to polyvinylidene difluoride (PVDF) or Nitrocellulose membranes. Antibodies specific to human/mouse α-synuclein (SynC20 recognizing the C terminal of both human and mouse α-synuclein, 1:1000, Santa Cruz; Syn-1 recognizing both human and mouse α-synuclein encoding amino acids 1-100, 1:1000, BD Biosciences, San Diego) [35] and β-actin (1:5000, Sigma) as loading control were used in this study. Horseradish peroxidase conjugated secondary antibodies were from Jackson ImmunoResearch. Signals were visualized by enhanced chemiluminescence development (Pierce, Rockford, IL) and quantified by imageJ software (NIH).

### COX-1 inhibition

For COX-1 inhibitor treatment, mutant mice and control littermates at 2 months of age were administrated with SC-560 (30 mg/kg; Cayman Chemical, Ann Arbor, MI, USA) or vehicle (40% dimethyl sulfoxide in 0.1 M phosphate buffer, pH 7.4) through intraperitoneal (IP) injection once a day for 7 days [34].

### Quantitative real-time PCR array

RNA was harvested using Qiagen RNeasy mini kit and converted into first-strand cDNA using RT^2 ^First Strand Kit (SuperArray Bioscience Corporation). Quantitative real-time PCR was performed using an ABI Prism 7900HT Fast Detection System (Applied Biosystems).

### Statistical Analysis

Statistical analysis was performed using the Graphpad Prism 5 (Graphpad Software Inc. La Jolla, CA). Data are presented as Means ± SEM. Statistical significances were determined by comparing means of different groups using t-test or ANOVA followed by Post Hoc Tukey HSD test, and presented as *p < 0.05, **p < 0.01, ***p < 0.001.

## Competing interests

The authors declare that they have no competing interests.

## Authors' contributions

XLG and HC designed the experiments and wrote the manuscript. XLG, CXL, CSX and LS performed the experiments. XL provided some materials. All authors have read and approved the final manuscript.

## Supplementary Material

Additional file 1 **PD-related human A53T *α-synuclein *was selectively expressed in the astrocytes of *A53T *transgenic mice**. **(A) **Western blot analysis reveals the expression level of exogenous α-syn in asymptomatic *A53T *mice. Protein extracts (5 μg) from the hippocampus were diluted by 1, 2, 4, 8, 16, 32 and 64-fold and equal volume of diluted samples was subjected to Western blot with a human/mouse specific α-syn antibody, α-syn (C20). **(B-E) **To determine the expression pattern of tTA under the GFAP promoter, *GFAP-tTA *mice were crossbred with *tetO-HIST1H2BJ/GFP *to yield *GFAP-tTA/tetO-GFP *mice. *HIST1H2BJ/GFP *is located in the nucleus. Brain sections of *GFAP-tTA/tetO-GFP *mice were stained with NeuN (neuronal marker, **B**), Iba1 (microglia marker, **C**), OSP (oligodentrocyte specific protein, **D**), and GFAP (marker for astrocytes, **E**). Scale bars: 20 μm. Click here for file

Additional file 2 **Progressive reduction of body weight and spontaneous locomotor activities in *A53T *mice**. **(A) **Representative photos of *A53T *mice and age-matched littermate controls under anesthetized condition. **(B) **Bar graph shows the body weight of *A53T *mice and control littermates at 1 and 2 months of age. ***p < 0.001. **(C-D) **Bar graphs depict the fine movement **(C) **and rearing activities **(D) **of *A53T *mice and control littermates at 1 and 2 months of age. *p < 0.05, ***p < 0.001. **(E) **Bar graph displays results from Rotarod test of *A53T *mice and littermate controls. Latency to fall was recorded at 1 and 2 months of age. **(F) **Body weight curves of *A53T *mice and littermates from 1 to 3 months of age. The body weight of *A53T *mice and littermates was measured twice a week. Concurrent with the abnormal motor behavior symptoms, the body weight of *A53T *mice was dropped continuously. Once the mice were unable to feed themselves (usually, body weight was dropped by 30%), they were sacrificed for histology and biochemistry study. Click here for file

Additional file 3 **The TreadScan Gait Analysis System (Clever Sys, Reston, VA) was used to record the gait information of *A53T *mice.** Each mouse was placed on the belt of treadmill unit. The movement of each paw was recorded with the treadmill running at 17 cm/s, 5 cm/s and 2 cm/s, respectively [54]. A representative control *nTg *mouse was running on the treadmill with the speed set at 17 cm/s. The later part of movie was replayed four times slower than the first part to better visualize the placement of each paw on the belt. Click here for file

Additional file 4 **Symptomatic *A53T *mice were only able to run at the speed of 5 cm/s.** The first mouse showed paralysis in both forelimbs. The second mouse displayed paralysis in the left forelimb and in both hindlimbs. Click here for file

Additional file 5 ***A53T *mice developed rapid progression of paralysis in four limbs.** This mouse was able to run at the speed of 5 cm/s on postnatal day 65 (P65), but 2 cm/s on P70. The right forelimb started to show paralysis on P71 and then the left forelimb was affected on P73. Click here for file

Additional file 6 **Behavior analysis of *A53T *mice treated with doxycycline (DOX) from embryonic stages to postnatal day 21 (P21)**. **(A) **Diagram outlines the treatment of *A53T *mice with DOX. DOX-containing mouse feed (200 mg/kg, Bioserv, Frenchtown, NJ) was provided to breeding pairs and young pups till the pups were weaned at postnatal day 21. The new weanlings were then switched to regular feed. **(B) **Dot plot shows the change of body weight of *A53T *mice and littermate controls after the stop of DOX treatment at P21. **(C-E) **Dot plots show the results of Open-field test of *A53T *and control mice after the stop of DOX treatment at P21. The spontaneous ambulatory (C), fine movement (D), and rearing activities (E) were quantified. **(F) **Dot plot depicts the performance of *A53T *and control mice on Rotarod test after the stop of DOX-treatment at P21. **(G) **Line graph shows the onset of paralysis of *A53T *mice after the stop of DOX-treatment at P21. Click here for file

Additional file 7 **Increase of GFAP and Iba1 expression in *A53T *mice**. **(A-B) **Bar graph shows quantitative RT-PCR analysis of *Gfap *and *Iba1 *transcripts expressed in the brainstem and cortex of *A53T *mice and age-matched *GFAP-tTA *mice (n = 3 per genotype). BS, brainstem; Ctx, cortex; 1 M, 1 month of age; 2.5 M, 2.5 months of age; A53T*, symptomatic *A53T *mice. *p < 0.05, and ***p < 0.001. **(C) **Western blots analysis of GFAP and Iba1 protein expression in the brainstem of *A53T *mice and littermate controls. **(D-E) **Bar graphs show the quantification *GFAP *(D) and *Iba1 *(E) expression in the brainstem, spinal cord, and cerebral cortex (n = 3 per genotype) of *A53T *and control mice. *p < 0.05, **p < 0.01 and ***p < 0.001. Click here for file

Additional file 8 **Activated microglia surrounded dopaminergic and spinal motor neurons**. **(A-F) **Representative images show double immunofluorescent labeling of Iba1 (green) with TH (red) and SMI32 (red) in the SNpc (**A-C**) and spinal cord (SC) (**D-F**) of symptomatic *A53T *mice. Scale bar: 20 μm. Click here for file

Additional file 9 **Quantification of cortical and striatal neurons remained in symptomatic *A53T *mice**. (A), Bar graph depicts the numbers of TH-positive dopaminergic neurons in the substantia nigra pars compacta (SNpc) and ventral tegmentum area (VTA) in *nTg *and *A53T *mice at 1 month of age. N = 3 per genotype.(B), Bar graph depicts the numbers of NeuN-positive neurons in the cerebral cortex and striatum of symptomatic *A53T *mice and age-matched *nTg *littermates estimated by unbiased stereological methods. N = 3 per genotype. Click here for file

Additional file 10 **Reactive astrocytes in the brainstem and substantia nigra pars compacta of *A53T *lower expresser mice (*A53T-A8*)**. **(A, C, E) **Representative images of GFAP (red) and Iba1 (green) staining show mild astrocytosis in the brainstem and SNpc but not in the cerebral cortex of 12-month old *A53T-A8 *mice compared to littermate *nTg *mice. **(B, D, F) **High magnification views of (**A, C, E**) reveal the morphology of astrocyte and microglia in control *nTg *and *A53T *mice at 12 month of age. Scale bars: 50 μm(**A, C, E**); 20 μm **(B, D, F)**. Click here for file
