# Supplementary figures and images for: Astrocytic expression of Parkinson's disease-related A53T α-synuclein causes neurodegeneration in mice
Source: Mol Brain. 2010 Apr 21;3:12. doi: 10.1186/1756-6606-3-12 (PMC2873589; doi:10.1186/1756-6606-3-12)

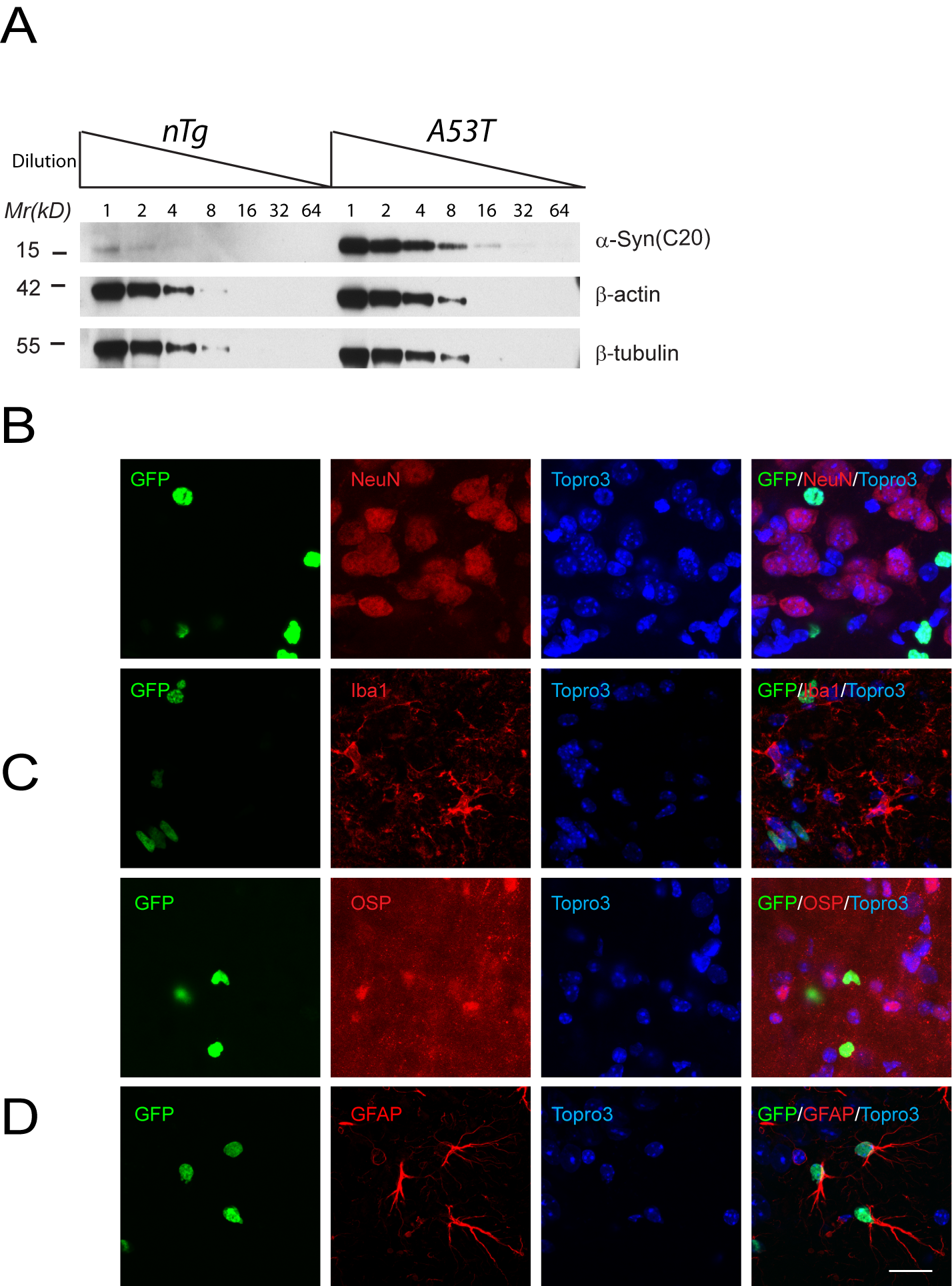

Supplement: Additional file 1 — PD-related human A53T α-synuclein was selectively expressed in the astrocytes of A53T transgenic mice. (A) Western blot analysis reveals the expression level of exogenous α-syn in asymptomatic A53T mice. Protein extracts (5 μg) from the hippocampus were diluted by 1, 2, 4, 8, 16, 32 and 64-fold and equal volume of diluted samples was subjected to Western blot with a human/mouse specific α-syn antibody, α-syn (C20). (B-E) To determine the expression pattern of tTA under the GFAP promoter, GFAP-tTA mice were crossbred with tetO-HIST1H2BJ/GFP to yield GFAP-tTA/tetO-GFP mice. HIST1H2BJ/GFP is located in the nucleus. Brain sections of GFAP-tTA/tetO-GFP mice were stained with NeuN (neuronal marker, B), Iba1 (microglia marker, C), OSP (oligodentrocyte specific protein, D), and GFAP (marker for astrocytes, E). Scale bars: 20 μm. [file 1756-6606-3-12-S1.TIFF]

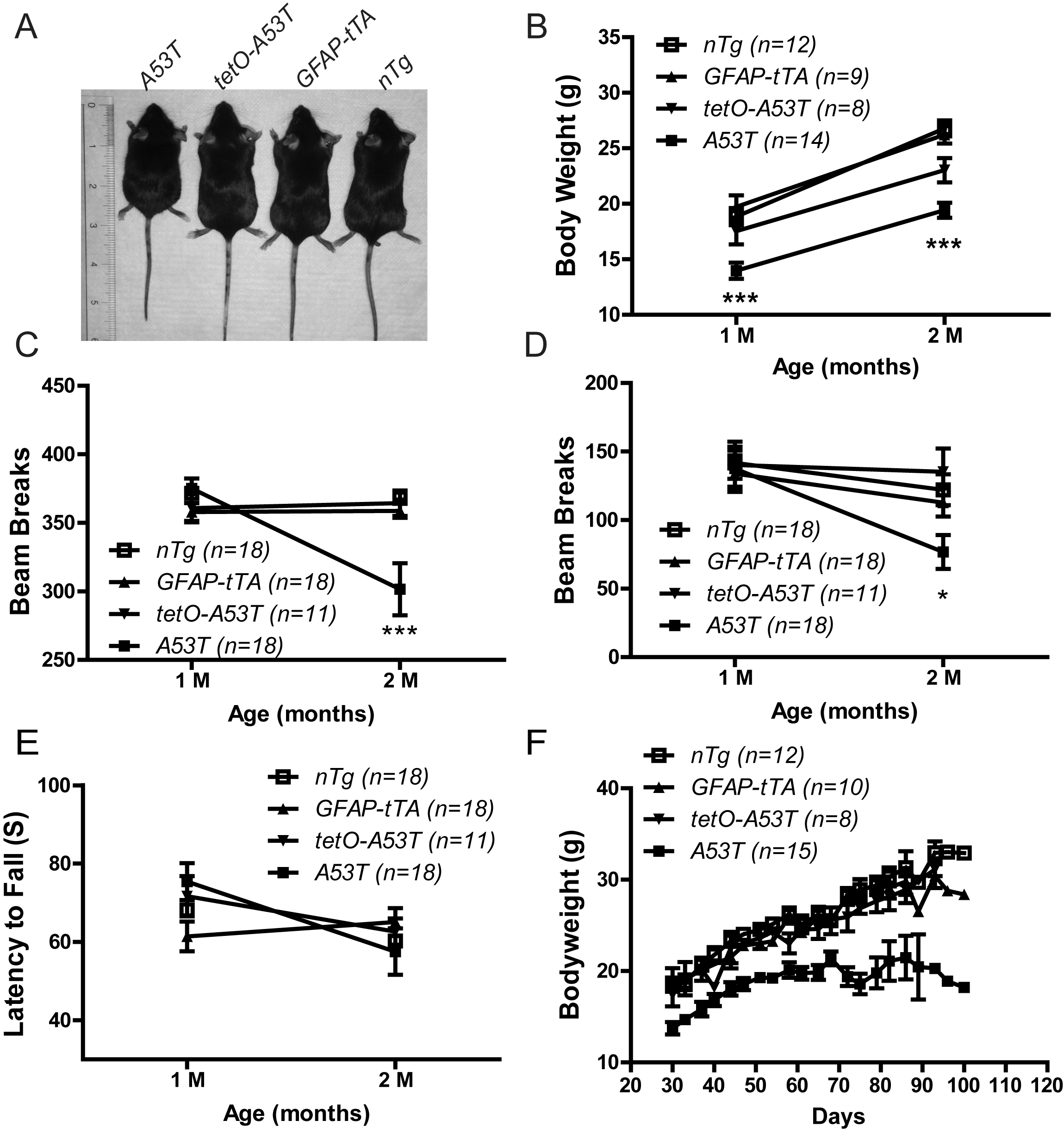

Supplement: Additional file 2 — Progressive reduction of body weight and spontaneous locomotor activities in A53T mice. (A) Representative photos of A53T mice and age-matched littermate controls under anesthetized condition. (B) Bar graph shows the body weight of A53T mice and control littermates at 1 and 2 months of age. ***p < 0.001. (C-D) Bar graphs depict the fine movement (C) and rearing activities (D) of A53T mice and control littermates at 1 and 2 months of age. *p < 0.05, ***p < 0.001. (E) Bar graph displays results from Rotarod test of A53T mice and littermate controls. Latency to fall was recorded at 1 and 2 months of age. (F) Body weight curves of A53T mice and littermates from 1 to 3 months of age. The body weight of A53T mice and littermates was measured twice a week. Concurrent with the abnormal motor behavior symptoms, the body weight of A53T mice was dropped continuously. Once the mice were unable to feed themselves (usually, body weight was dropped by 30%), they were sacrificed for histology and biochemistry study. [file 1756-6606-3-12-S2.TIFF]

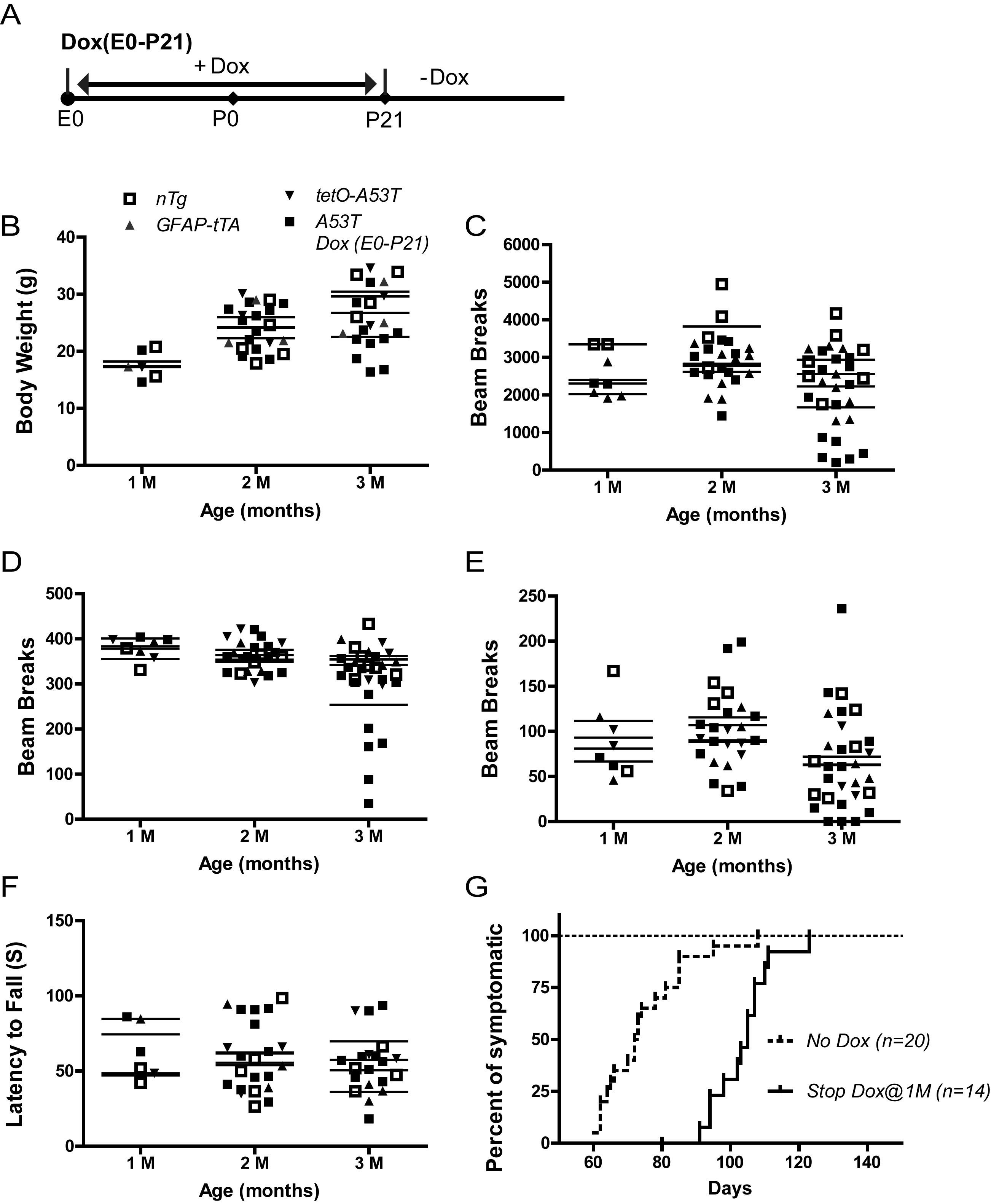

Supplement: Additional file 6 — Behavior analysis of A53T mice treated with doxycycline (DOX) from embryonic stages to postnatal day 21 (P21). (A) Diagram outlines the treatment of A53T mice with DOX. DOX-containing mouse feed (200 mg/kg, Bioserv, Frenchtown, NJ) was provided to breeding pairs and young pups till the pups were weaned at postnatal day 21. The new weanlings were then switched to regular feed. (B) Dot plot shows the change of body weight of A53T mice and littermate controls after the stop of DOX treatment at P21. (C-E) Dot plots show the results of Open-field test of A53T and control mice after the stop of DOX treatment at P21. The spontaneous ambulatory (C), fine movement (D), and rearing activities (E) were quantified. (F) Dot plot depicts the performance of A53T and control mice on Rotarod test after the stop of DOX-treatment at P21. (G) Line graph shows the onset of paralysis of A53T mice after the stop of DOX-treatment at P21. [file 1756-6606-3-12-S6.TIFF]

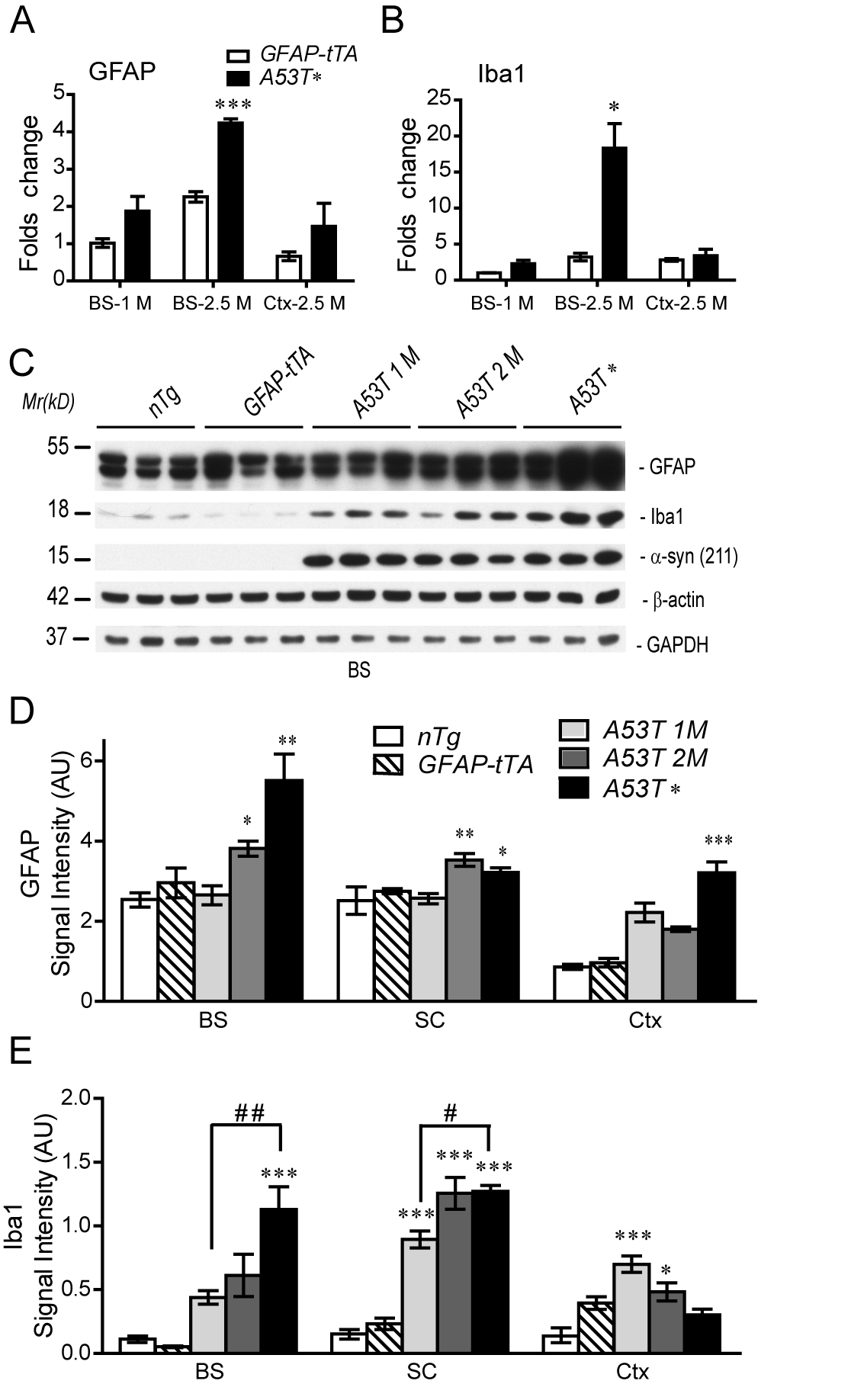

Supplement: Additional file 7 — Increase of GFAP and Iba1 expression in A53T mice. (A-B) Bar graph shows quantitative RT-PCR analysis of Gfap and Iba1 transcripts expressed in the brainstem and cortex of A53T mice and age-matched GFAP-tTA mice (n = 3 per genotype). BS, brainstem; Ctx, cortex; 1 M, 1 month of age; 2.5 M, 2.5 months of age; A53T*, symptomatic A53T mice. *p < 0.05, and ***p < 0.001. (C) Western blots analysis of GFAP and Iba1 protein expression in the brainstem of A53T mice and littermate controls. (D-E) Bar graphs show the quantification GFAP (D) and Iba1 (E) expression in the brainstem, spinal cord, and cerebral cortex (n = 3 per genotype) of A53T and control mice. *p < 0.05, **p < 0.01 and ***p < 0.001. [file 1756-6606-3-12-S7.TIFF]

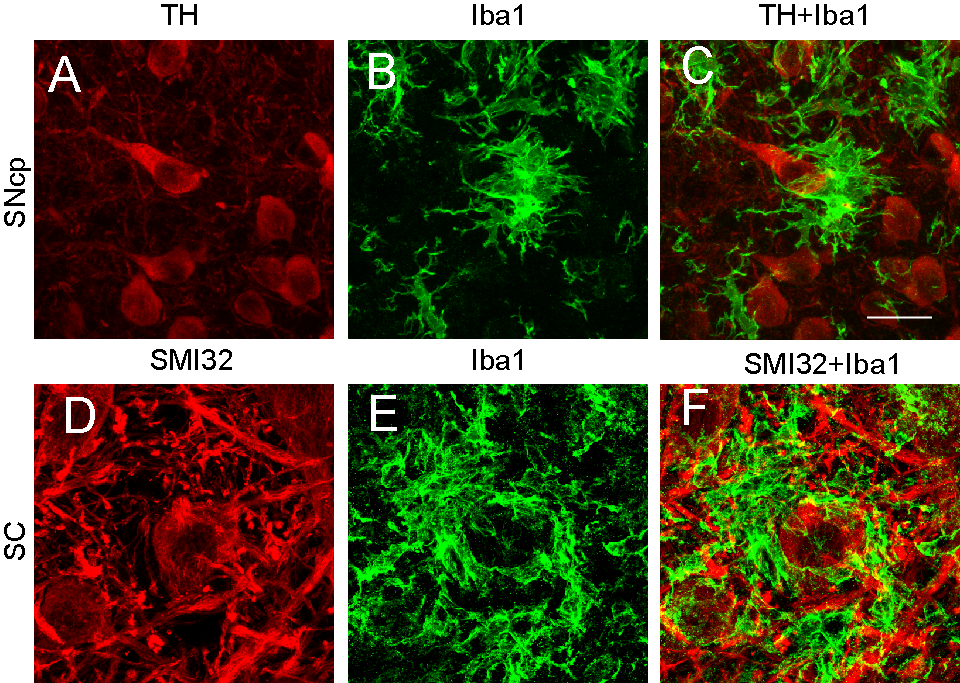

Supplement: Additional file 8 — Activated microglia surrounded dopaminergic and spinal motor neurons. (A-F) Representative images show double immunofluorescent labeling of Iba1 (green) with TH (red) and SMI32 (red) in the SNpc (A-C) and spinal cord (SC) (D-F) of symptomatic A53T mice. Scale bar: 20 μm. [file 1756-6606-3-12-S8.TIFF]

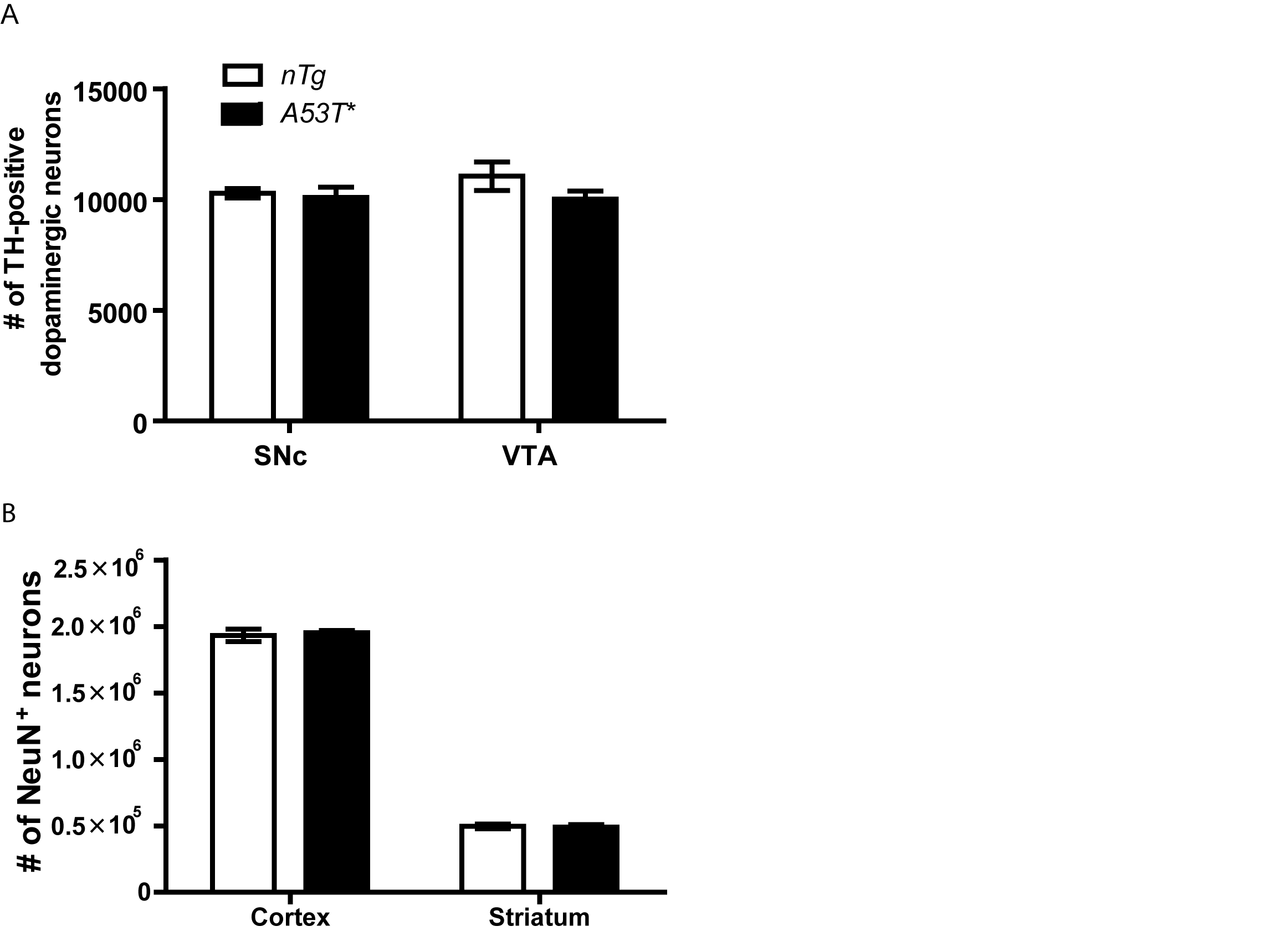

Supplement: Additional file 9 — Quantification of cortical and striatal neurons remained in symptomatic A53T mice. (A), Bar graph depicts the numbers of TH-positive dopaminergic neurons in the substantia nigra pars compacta (SNpc) and ventral tegmentum area (VTA) in nTg and A53T mice at 1 month of age. N = 3 per genotype.(B), Bar graph depicts the numbers of NeuN-positive neurons in the cerebral cortex and striatum of symptomatic A53T mice and age-matched nTg littermates estimated by unbiased stereological methods. N = 3 per genotype. [file 1756-6606-3-12-S9.TIFF]

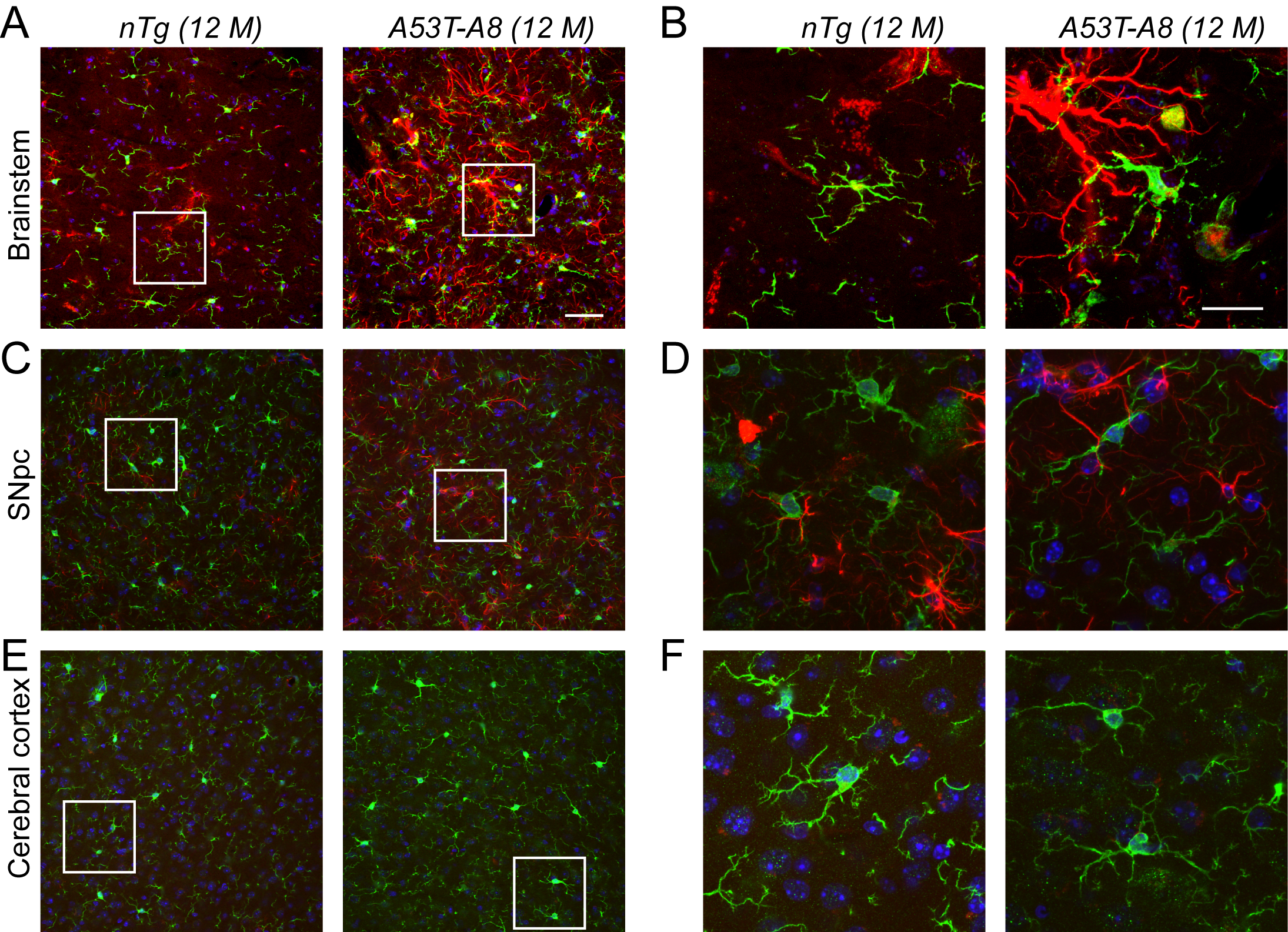

Supplement: Additional file 10 — Reactive astrocytes in the brainstem and substantia nigra pars compacta of A53T lower expresser mice (A53T-A8). (A, C, E) Representative images of GFAP (red) and Iba1 (green) staining show mild astrocytosis in the brainstem and SNpc but not in the cerebral cortex of 12-month old A53T-A8 mice compared to littermate nTg mice. (B, D, F) High magnification views of (A, C, E) reveal the morphology of astrocyte and microglia in control nTg and A53T mice at 12 month of age. Scale bars: 50 μm(A, C, E); 20 μm (B, D, F). [file 1756-6606-3-12-S10.TIFF]
